# Auditory Steady-State Response and Hearing Impairment in Survivors of Childhood Bacterial Meningitis in Luanda, Angola

**DOI:** 10.3390/jcm12082842

**Published:** 2023-04-13

**Authors:** Mariia Karppinen, Emilie Rugemalira, Okko Savonius, Manuel Leite Cruzeiro, Antti Aarnisalo, Topi Jutila, Tuula Pelkonen

**Affiliations:** 1 Department of Otorhinolaryngology–Head and Neck Surgery, University of Helsinki, Helsinki University Hospital, 00290 HUS Helsinki, Finland; 2 Children’s Hospital, Pediatric Research Center, University of Helsinki, Helsinki University Hospital, 00290 HUS Helsinki, Finland; 3 David Bernardino Children’s Hospital, Rua Amilcar Cabral, Maianga, Luanda, Angola

**Keywords:** sensorineural hearing loss, deafness, unilateral hearing loss, objective audiometry, child, low- and middle-income country, bacterial meningitis

## Abstract

Survivors of childhood bacterial meningitis (BM) often develop hearing impairment (HI). In low- and middle-income countries (LMICs), BM continues to be a significant cause of hearing disability. We assessed hearing among BM survivors using auditory steady-state responses (ASSR), providing frequency-specific estimated audiograms, and examined whether ASSR would provide a greater understanding of BM-induced HI. Survivors from two prospective BM trials (ISRCTN62824827; NCT01540838) from Luanda Children’s Hospital were examined in a follow-up visit with a median duration of 26 months after BM. The hearing of 50 BM survivors and 19 control children was evaluated using ASSR and auditory brainstem response (ABR) after interview and neurological and otorhinolaryngological examinations. The median age of survivors was 80 (IQR 86) months. We diagnosed HI (better ear hearing ≥ 26 dB) in 9/50 (18%) children. Five of the fifty survivors (10%) and 14/100 ears (14%) had profound HI (>80 dB). Severe-to-profound HI affected all frequencies steadily, affecting only the ears of BM survivors (18/100 vs. 0/38, *p* = 0.003). When looking only at the severely or profoundly affected ears, young age, low Glascow coma score, pneumococcal aetiology, and ataxia were associated with a worse hearing outcome.

## 1. Introduction

In low- and middle-income countries (LMICs), survivors of childhood bacterial meningitis (BM) often suffer from multiple sequelae, of which sensorineural hearing impairment (HI) is the most common [1]. BM is an infection of particularly early childhood. Despite the decrease in incidence over recent decades, the disease is still a significant cause of mortality and morbidity in children under the age of five years old [2]. In Africa, the high share of the global meningitis burden stems from the young age of the population, the high rate of endemic disease, and frequent epidemics [3,4].

After BM, multiple and major sequelae occur, of which HI is the most common, reported in up to 34% of cases and ranging from mild to profound and unilateral to bilateral, typically occurring early on in the disease [1]. In BM-related HI, labyrinthitis damages the hair cells [5], leading to spiral ganglion neuron loss, connective tissue obliteration, and possible fibrosis and ossification of the cochlea [6,7]. Illness-, host-, and treatment-related factors contribute to the risk of HI [8]. In areas of high BM incidence, BM-related HI remains a significant cause of avoidable HI [9].

Pure-tone play audiometry and pure-tone audiometry are measurements of hearing requiring the child’s cooperation, typically suitable for a child beyond infancy and over five years of age, respectively. In LMICs, pure-tone audiometry is the most accessible method, while the testing of young children who are most often affected by BM and HI is often lacking [10]. Additionally, a significant number of BM survivors manifest neurological abnormalities and cognitive disabilities, making it impossible for them to participate in behaviour-dependent tests. The early diagnosis of HI is crucial in prelinguistic and speech learning ages due to the central auditory processing. In LMICs, the lack of audiologists, ENT specialists, and rehabilitation services expose survivors to a risk of disability [11]. The chain of treatment from recognising HI to high-level rehabilitation services is weak. Recognition of HI is challenging for parents amongst the youngest (often prelingual) children [12], while awareness of BM-induced HI among physicians may be limited as well.

The hearing evaluation of young children and disabled individuals who are unable to participate in traditional behavioural tests relies on objective methods. Auditory steady-state response (ASSR) and auditory brainstem response (ABR) are both evoked electric potentials created by acoustic stimuli, and are used to estimate hearing sensitivity. Rapid and repeated auditory stimuli evoke ASSR, which is then detected and analysed using statistical methods to predict the presence or absence of a response that is analysed in the frequency domain [13]. The technique allows the simultaneous binaural testing of auditory thresholds.

The frequency-specific narrow-band CE-Chirp^®^ stimuli are sound stimuli developed to reduce delay related to the cochlear travelling wave. The responses are recorded using electrodes placed on the skull while background noise and biological electric activity are filtered out using a variety of techniques. ASSR has shown feasibility and accuracy in estimating hearing thresholds in young children [14]. While ABR correlates with behavioural thresholds [15], it may rely on interpretation, although automated testing has become available for ABR applications as well. The advantage of ASSR is the use of a band of four frequencies and the ability to create an estimated audiogram of hearing.

We aimed to test the hearing of patients via ASSR to acquire estimated audiograms to provide accurate counselling for those with hearing loss, and to examine whether ASSR would provide more data on BM-induced HI. To our knowledge, ASSR has not been described in BM before.

## 2. Materials and Methods

Survivors from two prospective BM treatment trials (ISRCTN62824827; NCT01540838) [16,17] from Luanda Children’s Hospital (university-level referral hospital and the only public children’s hospital in the capital of Angola) were called for follow-up visits in January 2017. The details of the studies have previously been published. On arrival at the hospital, the attending paediatrician carried out clinical examination and performed a spinal tap on all children. Cerebrospinal fluid samples were examined via routine cytological and biochemical testing and causative bacteria were identified via the culture, polymerase chain reaction, or latex agglutination test.

We examined the hearing of 50 survivors using ASSR and ABR and the Eclipse platform (Interacoustics, Middelfart, Denmark). A control group of 19 children was also included. The controls were healthy children who had no history of BM visiting the hospital during our study. They were siblings to the BM survivors (N = 11), or healthy children attending the hospital to receive vaccinations (N = 8) for rabies.

The families were interviewed using a structured questionnaire in their native language. Our study paediatrician (TP) carried out a clinical and neurological examination on each child. Before the hearing evaluation, the study doctors, residents in paediatrics and otorhinolaryngology, performed a pneumatic otoscopy for each patient after cleaning the ear canal with cotton swabs when necessary. In case of any uncertainty of the ear status, a crosscheck was carried out. Concomitant ear pathology was not defined as an exclusion criterion, as all participants deserved an evaluation of their hearing. Prior to the study, the study doctors received detailed training to perform ASSR and ABR recordings.

**Figure 1:**
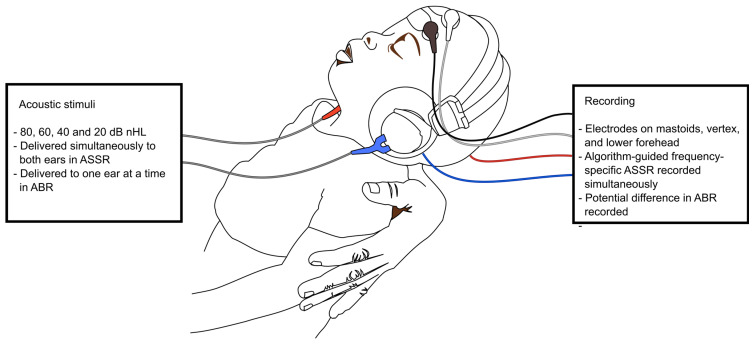
Hearing testing using auditory steady-state response (ASSR) and auditory brainstem response (ABR).

The hearing evaluation was carried out in a quiet hospital room without other electronic devices turned on, except for the study computers. Between patients, the room was cooled down with an air conditioner. The electrodes were placed on cleaned and rubbed skin on the mastoids (negative electrodes), vertex (positive electrode), and low forehead (ground) for the 2-channel ASSR and ABR recording. Before stimulation, acceptable skin impedance was verified. Infants and young children were held in a comfortable position on the parent’s lap and were encouraged to fall asleep after bottle- or breastfeeding; older participants lay in bed, and were instructed to relax, close their eyes, and stay still, and were allowed to fall asleep. General anaesthesia was not necessary in any case. Hearing testing and electrode placement are illustrated in Figure 1.

The narrow-band CE-Chirp^®^ stimuli were delivered binaurally using headphones with intensities of 80, 60, 40, and 20 dB nHL (normal hearing level) at a rate of 90 Hz in ASSR. In ABR, the stimuli were delivered to the ears separately with the contralateral side, receiving 20 dB lower masking using the above-mentioned intensities. Recording used the ASSR and ABR modules of the Eclipse platform. In ASSR, the algorithm-guided frequency-specific recording was stopped when the level of significance for a response was reached or if no response was found during six minutes of recording. The thresholds were calculated using proprietary algorithms. In ABR, the in-built algorithm gave a pass/refer result based on a reproducible wave V. The intensities were lowered or raised until the lowest threshold was reached.

For ASSR, the collected data were estimated thresholds in dB at 0.5, 1, 2, and 4 kHz. For an overall estimation of hearing, a mean of thresholds was calculated. WHO hearing loss grades were used to categorise the patients into groups of mild (26–40 dB nHL), moderate (41–60 dB nHL), severe (61–80 dB nHL), and profound HI (>80 dB nHL). A hearing threshold of ≤25 dB nHL indicated normal hearing. A better ear hearing threshold of >30 dB nHL was defined as disabling HI, according to the WHO definition [18,19]. If a patient did not hear the stimulus at 80 dB nHL, an estimated value of 81 dB was used.

The ethics committee of Hospital Pediátrico David Bernardino approved both treatment trials, including amendments for the current follow-up study. Before inclusion to the original trials, the child’s guardian gave informed consent. The follow-up evaluation was voluntary for all families. Based on paediatric and hearing evaluations, counselling and appropriate references were given in line with the local health system. During the study, local residents were trained in pneumatic otoscopy and audiological testing.

We analysed the data using SPSS 28.0 for MacOS (IBM Corp., Armonk, NY, USA) and used descriptive methods to present the findings. We used Fisher’s exact test to compare dichotomous categorical variables and the nonparametric Mann–Whitney *U* test to compare medians, and calculated Spearman rho values for correlations. The distribution of the data was graphically analysed, and the Shapiro–Wilk test was applied if needed. We applied statistical significance of *p* < 0.05.

## 3. Results

**Figure 2:**
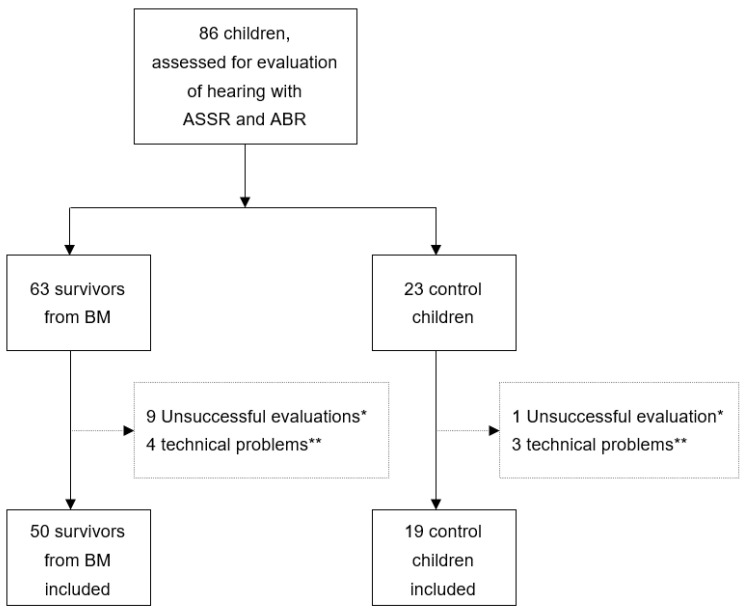
Flowchart of children from Angola included in the study. ASSR, auditory steady-state response; ABR, auditory brainstem response; BM, bacterial meningitis. * Evaluation unsuccessful due to restlessness (stopped or unable to proceed to recording; 8 BM patients could not proceed, and 1 BM patient and 1 control discontinued). ** Programme frozen or failure to save the recording.

The ASSR evaluation was attempted for 86 children (63 patients and 23 controls). Altogether, 69 children and 138 ears were successfully evaluated using ASSR and ABR (Figure 2). However, in three patients and two controls (and six ears), not all frequencies via ASSR were fully completed. In such cases, only the frequencies with successfully estimated ASSR thresholds were used in the evaluation.

During the follow-up study, the median age of the children was 80 (IQR 86.0) months. The follow-up study was performed 26 months (median, IQR 38) after BM. For controls, the median age was 85 (IQR 59) months. Eighteen out of fifty survivors (36%) were girls. Nine survivors were recruited from the first clinical trial [16], while forty-one survivors formed part of the second clinical trial [17].

### 3.1. Hearing Outcome in Patients and Controls

**Figure 3:**
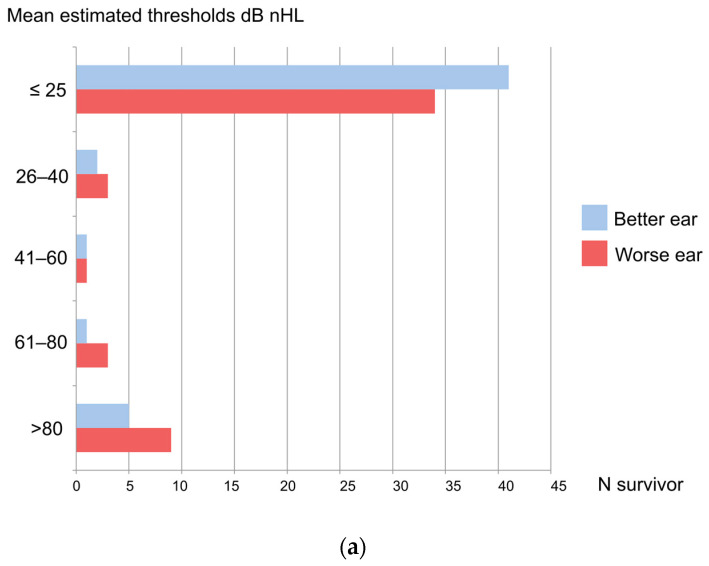

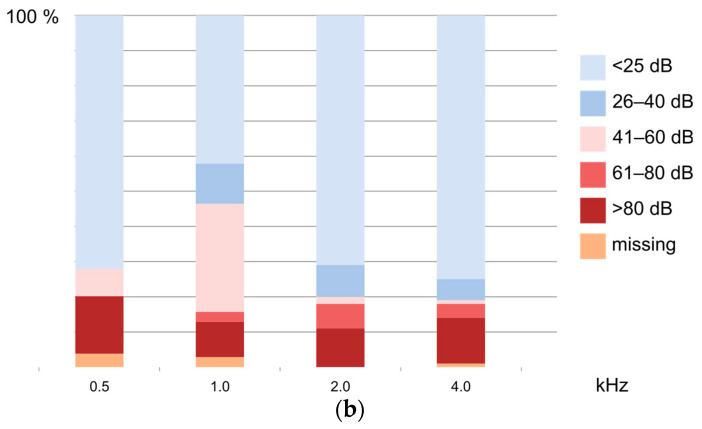
(**a**) Grades of hearing impairment from auditory steady-state responses (ASSR) after bacterial meningitis in Luanda, Angola; (**b**) ASSR results of ears of children having survived bacterial meningitis.

Out of 50 BM survivors, 9 (18%) had HI (better ear hearing ≥ 26 dB nHL). Disabling HI (>30 dB nHL) affected seven (14%) of the patients. Five of fifty patients (10%) and fourteen of one hundred ears (14%) were diagnosed as having profound HI, i.e., deafness (>80 dB nHL). Additionally, one (2%) suffered from severe HI (61–80 dB nHL), one (2%) suffered from moderate HI (41–60 dB nHL), and mild HI (26–40 dB nHL) occurred in two (4%) children. Any type of HI (of either ear) affected 16 (32%) of the patients. None of the controls had bilateral HI but 4 of 19 (21%) had unilateral HI. Table 1 displays the results of hearing evaluations using ASSR in BM patients and controls. Figure 3a displays the hearing loss grades of both worse and better ears in BM children.

The frequency-specific results of all ears (n = 100 in patients) are shown in Figure 3b. Severe-to-profound HI affected all frequencies steadily. In all ears, any HI of ≥ 26 dB was found in 25/100 patients and in 4/38 controls; *p* = 0.07. However, using a cut-off of 40 dB, HI was more common in patients’ than controls’ ears (20/100 vs. 1/38, *p* = 0.008). Similarly, using a cut-off of 60 dB, having severe or profound HI was significantly more common in patients’ vs. controls’ ears (18/100 vs. 0/38, *p* = 0.003), and profound HI of ears was more common with patients compared to controls (14/100 vs. 0/42, *p* = 0.01), as seen in Table 2.

The frequency-specific hearing threshold estimates of ears differed between patients and controls (*p* = 0.028; *p* = 0.058; *p* = 0.02; and *p* = 0.003 in Mann–Whitney *U* test, for frequencies 0.5, 1, 2, and 4 kHz, respectively). The mean ASSR threshold correlated with the ABR threshold in patients and controls (Spearman rho 0.76, *p* < 0.001 for better ear) and in the ears of patients and controls (Spearman rho 0.73, *p* < 0.001). The hearing grades obtained using ASSR and ABR are displayed in Table 1.

### 3.2. Hearing Outcome vs. Clinical Characteristics of BM and Findings during Follow-Up Evaluation

During admission to hospital due to suspected meningitis, one child had acute otitis media, two children had unknown middle ear status, and forty-seven had normal middle ear status. None of the children suffered from otorrhoea at the time of admission and one had otorrhoea during the hospital stay. At the time of the hearing evaluation, thirty-eight children (76%) had healthy ears in otoscopy while three children (6%) showed middle ear or tympanic membrane pathology on either ear (one had middle ear fluid, one had dry tympanic membrane perforation, and one had otorrhoea derived from perforated tympanic membrane (see Table 2 caption)). The hearing grades of these ears were normal hearing, normal hearing, and profound HI, respectively. The one BM child with one-sided otorrhoea presented with a bilateral > 80 dB hearing impairment. In nine children (18%), one or both of the tympanic membranes could not be studied due to cerumen. In 25 cases, the aetiology of BM could be defined: *Streptococcus pneumoniae*, *Neisseria meningitidis*, *Haemophilus influenzae* type b, and other causative agents in eight, seven, five, and five children, respectively.

Table 3 shows the comparison of severe-to-profound hearing-impaired ears vs. normal or milder HI in relation to patient characteristics, history of the disease, and hospital and laboratory findings. *S. pneumoniae* aetiology (8/12 vs. 8/38 ears, *p* = 0.01) and low haemoglobin (6.4 vs. 8 g/L, *p* = 0.018) were associated with a worse hearing outcome. At follow-up, ataxia (8/18 vs. 8/82, *p* = 0.038) and peripheral nystagmus (6/18 vs. 10/82, *p* = 0.033) were associated with HI.

Clinical suspicion of HI at follow-up was associated significantly with severe-to-profound HI. Two children were severely handicapped. One older child was attending school for hearing disabled children; another older child had dropped a class twice. These patients are described in greater detail in the Supplementary Table S1.

## 4. Discussion

HI is a frequent finding after BM, affecting 18% of BM survivors in the present study (better ear hearing ≥ 26 dB nHL). The proportion of disabling HI (>30 dB) in seven (14%) of the survivors, and severe-to-profound HI in six of the survivors (12%), with mostly deafness, is in line with the previous literature from high-BM-prevalence settings [1,16]. In our previous study, 12% of deafness was diagnosed after BM [16]. In a global meta-analysis, the risk of HI was reported to range from 3 to 17 % [1].

However, comparisons to previous data, specifically from similar settings, are challenging, as the youngest children are typically excluded or their testing does not rely on objective measures. Despite these shortcomings, a meta-analysis of BM sequelae in African children reported post-discharge HI of 2–34% [4]. A global meta-analysis highlighted the higher occurrence of sequelae and HI after BM in low-income countries [1]. ABR was used in a multi-centre Latin American study diagnosing a total of 27% of patients with HI after childhood BM with a 4% rate of deafness [20]. Our findings of 12% of severe-to-profound HI exceed this. Nonetheless, taking into account the higher mortality and sequelae rates of BM in Angola, we assume our data are not overestimated.

The proportion of any hearing impairment in ears did not differ significantly between BM survivors and controls. The mild-to-moderate HI of 7% in our survivors’ and 11% in our controls’ ears may be attributable in a few cases to impacted cerumen or other acquired causes of HI, such as measles, mumps, other infections, or sickle cell disease, all highly prevalent in Africa [9]. In fact, in Africa, a median of 7.7% of school-aged children suffer from HI of >25 dB in the better hearing ear, while a median of 6.6% experience disabling HI [21]. This might explain the HI in our control children. Here, the nonsignificant difference in any HI in ears between BM survivors and controls may be biased by the limited size of our control group. For BM children, a known HI was applied as an exclusion criterion in the original treatment trials. Regarding the tympanic membrane evaluation, in our study, effusion behind tympanic membrane was seldom diagnosed: one ear of a BM child and one control child bilaterally (2/69 children, 3%). This number may be underestimated; however, it falls into the ranges of other African studies reporting a prevalence of 1.5–3.6% of effusion in children [22,23]. One BM child had an otorrhoea trough perforated membrane and one control child had one-sided dry perforation.

As expected, a significant difference in the estimated ASSR frequencies between BM patients and controls was noted across the tested frequencies as the severe-to-profound HI occurred only in BM survivors. In addition, the ASSR showed that HI manifested similarly across the tested frequencies. In animal models of BM, the infection spreads from the subarachnoid space via the patent cochlear aqueduct to the labyrinth, resulting in gradual damage along the base to the axis [24]. Thus, the basal parts of the cochlea, responsible for high frequencies, are damaged first. With our survivors, however, no difference between the frequencies was noted in severe-to-profoundly affected ears. This is possibly due to connective tissue obliteration, fibrosis, and ossification of the cochlea after BM, which may take place soon after BM, especially in the case of *S. pneumoniae* [6,7,25]. Previously, lower frequencies of narrow-band CE-Chirp ASSR have shown less reliability than higher frequencies, although otherwise it has proven to be more accurate than traditional ASSR with good correlations with behavioural pure tone thresholds [26].

The success rate for performing both ASSR and ABR was quite high: 69/86 (80%) amongst the children included in the study (Figure 2). In 69 children, the ASSR and ABR were successfully measured and estimated audiograms and ABR results were provided following the protocols of the manufacturer. General anaesthesia or sedatives were not used, although no hospital resources were allocated for such. Despite the age span of these 69 children (median 85, range 8–197 months), hearing evaluation was mostly successful. The children were calmed down with their parents’ care, patience, and breast- or bottle-feeding in the case of younger children. Many of the children were allowed to fall into natural sleep with the parent’s guidance, while the testing environment was leisured and comfortable. The ASSR-estimated audiograms provided practical certainty to counsel and refer the children with HI and/or their caregivers. We did not measure the time of ASSR and ABR procedures, but the automated ABR was performed much faster and correlated well with the ASSR findings, and may be suitable for otherwise challenging conditions with limited health care resources.

When looking only at the severely or profoundly affected ears, young age, low Glasgow coma score, *S. pneumoniae* aetiology, and low blood haemoglobin were associated with worse hearing outcomes. These have all been previously described as being BM-related risk factors for unfavourable hearing outcomes in LMIC settings [20,27], and likely reflect the severity of the infection and concomitant inflammation response. Low haemoglobin has recently been shown to be a risk factor for sensorineural HI [28]. The underlying mechanism remains unclear; however, as the labyrinthine artery exclusively supplies the cochlea, low haemoglobin may add to its ischemic vulnerability.

In LMICs, the presenting condition of a child during BM has been the determinant factor predicting mortality and severe sequelae overruling many other factors such as the aetiology [20,27]. The meta-analyses, however, do present a clear tendency of more HI in *S. pneumoniae* and *H. influenzae* type b BM, while *N. meningitidis* more often leaves hearing undamaged [1,4]. Similarly, in our study, *S. pneumoniae* was associated with a severe-to-profound HI of ears. Aetiology, however, should not determine the need for auditory testing after BM.

The limited sample size likely affected our results, as we did not find associations with other risk factors reported in African studies, such as convulsions, associated cranial nerve findings, low blood leukocyte count, and high CSF protein levels [29,30]. As seen before, ataxia was associated with HI [31], while nystagmus, which was associated with HI in the present study, likely reflects concomitant peripheral vestibular damage after BM. Multiple severe sequelae were associated with HI in our patients as well [1].

This study has several limitations. We could not recruit all of the survivors of our studies, which may have biased our study population. Additionally, 20% of the children intended for hearing evaluation could not participate, due to either not being able to calm down for the audiometry or technical problems which arrested the testing. In addition, our control group was rather small, which may also affect some of our comparisons stated above. The resources enabled us to examine hearing using only ASSR and ABR.

The equipment to clean the ear canal was not optimal, as in 12% of all ears (BM children and controls) cerumen blocked the view in otoscopy even though the median hearing did not statistically differ between ears with or without cerumen. The ear status was normal with mild-to-moderate HI in all except three ears (in one control ear with effusion, mean of 26 dB ASSR-threshold, and two survivors with cerumen and means of 43 and 74 dB ASSR-thresholds). The very limited resources hampered the possibility to clear the ear canal as cotton swabs were the only available instruments. No suction devices for ear wax removal were available; in addition, the children’s hospital lacks a tympanometer and an otomicroscope. These are limitations that have likely affected our results and need to be taken into account when interpreting our results; however they might reflect the reality in many resource-limited settings.

The limited capacity to meet the demands of rehabilitation in cases of severe-to-profound HI was demonstrated with our patients, as none of them had access to hearing aids or cochlear implants, which are lacking in Luanda. However, to tackle these inequalities in Africa, WHO has launched resolutions addressing ear and hearing care by establishing training programmes, improving access to affordable and cost-effective hearing technologies, and promoting alternative communication methods [32].

## 5. Conclusions

HI is a major sequela after BM, with severe-to-profound HI distinguishing the patients from controls and occurring in 6/50 (12%) children and in 18/100 (18%) ears. This HI occurred across all ASSR frequencies and did not differ from the ABR results. A young age at time of BM, low Glasgow coma score at presentation, pneumococcal aetiology, and ataxia were all associated with HI. As BM-induced HI often affects children of prelingual age, prompt diagnosis and rehabilitation matters are crucial. The need for accessible ear and hearing care and education after BM in LMIC settings is significant.

## Figures and Tables

**Table 1:** Estimated hearing grades (ASSR and ABR) of children having survived bacterial meningitis and controls in Luanda, Angola.

	BM Survivor N = 50		Control N = 19		*p* Value
Median age in months, median (IQR)	80 (48–134)		85 (74–133)		0.29
Female sex, n (%)	18 (36)		9 (47)		0.42
Hearing loss grades	ASSR	ABR	ASSR	ABR	
Normal hearing, n (%)	41 (82)	36 (72)	19 (100)	19 (1)	
Mild HI, n (%)	2 (4)	7 (14)	0	0	
Moderate HI, n (%)	1 (2)	1 (2)	0	0	
Severe HI, n (%)	1 (2)	1 (2)	0	0	
Profound HI, n (%)	5 (10)	5 (10)	0	0	
Disabling HI, n (%)	7 (14)	n/a	0	n/a	
Any HI of either ear, n (%)	16 (32)	23 (46)	4 (21)	4 (21)	
Severe or profound HI of either ear, n (%)	12 (24)	12 (24)	0	0	

ASSR, auditory steady-state response; ABR, auditory brainstem response; BM, bacterial meningitis; HI, hearing impairment. Hearing grades refer to WHO hearing loss grades: ≤25 dB nHL normal hearing; 26–40 dB nHL mild HI; 41–60 dB nHL moderate HI; 61–80 dB nHL severe HI; >80 dB nHL profound HI [18,19]. *p* values obtained with Fisher’s exact test or Mann–Whitney *U* test as appropriate; significance with *p* < 0.05.

**Table 2:** Estimated hearing grades of ears in children having survived bacterial meningitis and controls in Luanda, Angola with ASSR.

Hearing Grade of Ears	Normal Hearing	Mild HI	Moderate HI	Severe HI	Profound HI	N All Ears
BM survivor, n	75	5	2	4	14	100
Controls, n	34	3	1	0	0	38
*p* value *		0.07	0.008	0.003	0.01	

ASSR, auditory steady-state response; BM, bacterial meningitis; HI, hearing impairment. Hearing grades refer to WHO hearing loss grades: ≤25 dB nHL normal hearing; 26–40 dB nHL mild HI; 41–60 dB nHL moderate HI; 61–80 dB nHL severe HI; >80 dB nHL profound HI [18,19]. The diagnosed ear pathologies in patients were as follows: 1 perforated ear with normal hearing, 1 ear with effusion and normal hearing, and 1 ear with otorrhoea and profound HI. The ear pathologies in controls were as follows: 2 ears with effusions, 1 with normal hearing, and 1 with mild HI, and 1 perforation with normal hearing. * *p* values obtained using binominal cut-off values of 25, 40, 60, and 80 dB in cross-tabulations between patients and controls, n = 138, Fisher’s exact test, significance with *p* < 0.05.

**Table 3:** Comparison of ears with estimated hearing impairment using ASSR in children having survived bacterial meningitis in Luanda, Angola.

	Ears with Severe-to- Profound HI N(%)/Median (IQR)	Ears with Normal-to- Moderate HI N(%)/Median (IQR)	*p* Value
	n = 18	n = 82	
Patients, history of the disease			
Female sex	7	29	0.79
Age in months during BM	12 (19)	39 (76)	0.012 *
Sick for how many days before admission?	4 (2)	5 (4)	0.24
Malnutrition	5/18	15/82	0.35
Seizures at home	12/16	48/78	0.398
Hospital findings			
Glasgow Coma Scale on admission	11 (7)	15 (4)	0.045 *
Coma	0/16	4/76	1.0
Seizures at hospital	14/18	46/80	0.18
Focal seizures	12/18	26/74	0.018 *
Focal neurological signs	6/18	22/80	0.77
Laboratory			
*S. pneumoniae* as causative agent	8/12	8/38	0.01 *
CSF leukocytes	400 (5383)	380 (2712)	0.54
CSF protein	190 (191)	147 (132)	0.46
CSF glucose	16 (35)	26 (39)	0.51
Plasma haemoglobin	6.4 (1.70)	8 (3.28)	0.018 *
Plasma CRP	161 (43)	156 (52)	0.073
HIV positivity	1/13	3/45	1.0
Sickle cell positivity	5/13	7/23	0.72
Follow-up findings			
Clinical suspicion of deafness	8/18	0/82	<0.001 *
Facial paresis, peripheral	1/18	1/82	0.33
Ataxia	8/18	8/82	<0.001 *
Nystagmus	6/18	10/82	0.038 *
Severe neurological sequelae (not HI)	4/18	4/82	0.033 *
Any neurological sequelae (not HI)	9/18	23/82	0.095

ASSR, auditory steady-state response; BM, bacterial meningitis; CSF, cerebrospinal fluid; HI, hearing impairment..Hearing grades refer to WHO hearing loss grades: ≤25 dB nHL normal hearing; 26–40 dB nHL mild HI; 41–60 dB nHL moderate HI; 61–80 dB nHL severe HI; >80 dB nHL profound HI [18,19]. *p* values obtained using Fisher’s exact test or Mann-Whitney *U* test as appropriate, * significance with *p* < 0.05.

## Data Availability

Data is contained within the article or Supplementary Material.

## Supplementary Materials

The following supporting information can be downloaded at https://www.mdpi.com/article/10.3390/jcm12082842/s1: Table S1: Patients with severe-to-profound hearing impairment of the better ear, and survivors of bacterial meningitis in Angola.
