# Supplementary material for: Auditory Steady-State Response and Hearing Impairment in Survivors of Childhood Bacterial Meningitis in Luanda, Angola
Source: J Clin Med. 2023 Apr 13;12(8):2842. doi: 10.3390/jcm12082842 (PMC10143662; doi:10.3390/jcm12082842)
Supplement: Supplementary file 1 [file jcm-12-02842-s001.zip › jcm-2276879-supplementary.pdf]

Table S1. Children with severe-to-profound hearing impairment, survivors of bacterial meningitis, in Luanda, Angola.

**Patient**	Mean ASSR thresholds R/L ear (dB nHL)	Age (months)	Language / verbal communication	Other
**1**	>80 / >80	29	No	
**2**	>80 / >80	16	No	
**3**	>80 / 79.5	14	No	Severe multiple disabilities
**4**	>80 / >80	141	Yes (writing)	Attending school for hearing disabled
**5**	>80 / >80	52	No	
**6**	>80 / >80	89	No	Severe multiple disabilities
